# Diffusive Gradients in Thin‐Films Technique as a Tool for Matrix Separation and Preconcentration for Sensitive Determination of Cd and Pb in Table Salt

**DOI:** 10.1002/fsn3.72040

**Published:** 2026-07-24

**Authors:** Marin Senila, Simion Bogdan Angyus

**Affiliations:** ^1^ National Institute for Research and Development of Optoelectronics INOE 2000 Research Institute for Analytical Instrumentation Cluj‐Napoca Romania

**Keywords:** DGT, food analysis, GFAAS, health risk, method validation, sample pretreatment, toxic elements

## Abstract

Herein, we developed a sensitive and accurate analytical methodology based on diffusive gradients in thin‐films (DGT) technique for matrix separation and preconcentration of Cd and Pb from table salt solution prior to their quantification by graphite furnace atomic absorption spectrometry (GFAAS). The methodology relies on the passive accumulation of the analytes from an aqueous table salt solution over a 24‐h period using a relatively simple and inexpensive tool. The linear accumulation of Cd and Pb during a 24 h DGT deployment was evaluated, with determination coefficients (*R* ^2^) of 0.9939 and 0.9887, respectively. The performance parameters of the proposed methodology were evaluated in accordance with the requirements specified in Commission Decision 2002/657/EC and Commission Regulations 2023/915/EU, 2011/836/EU, and 2007/333/EC. The limits of detection for Cd and Pb in salt samples were determined to be 38 and 81 μg kg^−1^, respectively. The method demonstrated trueness values ranging from 85% to 92%, precision expressed as relative standard deviations between 11.0% and 13.1%, and combined standard measurement uncertainties of 13.2% for Cd and 14.0% for Pb. These performance characteristics satisfy the minimum requirements mandated for analytical methods employed in official laboratories for the quantification of Cd and Pb in food matrices. The validated method was applied to the analysis of 10 types of refined and unrefined table salt from Romanian markets. In two samples, the Pb concentration exceeded the maximum level established by Commission Regulations 2023/915/EU. The proposed method shows good analytical potential and can be easily implemented in control laboratories.

## Graphical abstract

This study investigated the applicability of the diffusive gradients in thin‐films (DGT) technique for matrix separation and preconcentration of cadmium (Cd) and lead (Pb) from table salt solutions prior to their quantification by graphite furnace atomic absorption spectrometry (GFAAS). The performance parameters of the proposed methodology were evaluated in accordance with the requirements specified in Commission Decision 2002/657/EC and Commission Regulations 2023/915/EU, 2011/836/EU, and 2007/333/EC. These performance characteristics meet the minimum requirements mandated for analytical methods employed in official laboratories for the quantification of Cd and Pb in food matrices. The validated method was applied to the analysis of 10 types of refined and unrefined table salt from Romanian markets. The proposed method demonstrates strong analytical potential and can be readily implemented in control laboratories.

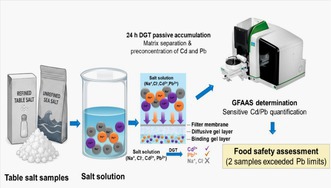

## 1 Introduction

Salt (sodium chloride) has been exploited and used in culinary practices since ancient times. Commonly identified as “table salt,” it provides two essential elements for the human body: sodium and chlorine (Mohammadi et al. 2025). Next to these elements, salt usually contains different trace elements. While some of those elements, such as Ca, Fe, Mg, K, Se Co, Cu, and Zn, are essential for human health, others like Cd, Pb, As, or Hg are considered nonessential elements and highly toxic, even at very low concentrations (Di Salvo et al. 2023; Sabala et al. 2024; Senila et al. 2025). Among these, Cd is classified as group I carcinogen to humans, with negative effects on pulmonary, renal, skeletal, cardiovascular, and reproductive systems (Nordberg et al. 2018). Lead exposure may cause neuronal damage, reduced intelligence quotient, dizziness, skeletal disorders, anemia, increased blood pressure, spontaneous abortion, and diminishing renal function, among other effects (Yang et al. 2025). The presence of Cd and Pb in the food ingredients is undesirable, and as a result, regulations have been established to limit the maximum allowable levels of these elements in foodstuffs, including table salt. In the European Union, Commission Regulation (EU) 2023/915 sets the for maximum levels in table salt for Cd and Pb as 0.50 and 1.0 mg kg^−1^ respectively. Other toxic elements regulated in table salt are As (0.50 mg kg^−1^) and Hg (0.10 mg kg^−1^).

The quantification of trace elements in salt table is a challenging analytical task due to their low concentration in a complex matrix. Trace element determination by electroanalytical methods, primarily anodic stripping voltammetry (ASV), offers a viable option due to its high sensitivity and relatively low instrumentation cost. However, potential drawbacks of these techniques include electrode fouling, interference from other metal ions, and reliance on meticulous calibration and sample preparation (Eftekhari et al. 2014; Xu et al. 2021; Yang et al. 2023). Atomic spectrometry techniques are among the most important analytical methods for trace element determination in various sample types. In general, they provide good accuracy and precision and are well suited for routine analysis. However, these methods can be affected by spectral interferences and, in the case of solid samples requiring dissolution prior to analysis, may not always provide sufficient sensitivity (Senila and Cadar 2025). In the case of As and Hg, hydride vapor generation and cold vapor generation can be applied, which can minimize or eliminate matrix interferences and improve the limits of detections (LODs) (Aksuner et al. 2011; Covaci et al. 2018; Sabala et al. 2024). Cd and Pb in food samples are primarily analyzed using spectrometric techniques, such as graphite furnace atomic absorption spectrometry (GFAAS), flame atomic absorption spectrometry (FAAS) inductively coupled plasma optical emission spectrometry (ICP‐OES), and inductively coupled plasma mass spectrometry (ICP‐MS) (Gao et al. 2025). In these techniques, the sample introduction systems typically accept only liquid samples, thus sample dissolution is required for solid sample analysis. In ICP‐based methods, the high quantity of dissolved salts creates matrix interferences such as signal suppression, signal drift, low signal to noise ratio, or even plasma collapse and nebulizer blockage. Determination of Cd and Pb by GFAAS technique in high‐salt solutions is also very challenging due to both spectral and nonspectral interference effects, as well as salt deposition on the graphite furnace, which significantly reduces its lifespan and increases the costs for consumables and maintenance (Senila 2024).

Sample dilution reduces some of these problems, but a too high dilution factor increases the limits of detection (LODs) which can make the analytical methodology inadequate for the determination of trace elements (Xiao et al. 2023). A practical method to lessen interferences and achieve reliable results is to use a separation technique for trace elements before spectrometric determination. To achieve this, complexing reagents, liquid–liquid extraction, solid‐phase extraction, or coprecipitation of trace metals were extensively studied (Amorim and Ferreira 2005; Soylak and Topalak 2015; Zhou et al. 2024). Still, these often involve many steps and chemicals, which increase the risk of sample contamination or analyte loss. Alternatively, a passive sampling technique, diffusive gradients in thin films (DGT), can provide matrix separation and preconcentration of metal cations. This technique has been primarily developed and used to measure metals in aquatic environments (Davison and Zhang 1994), and its applicability was then extended to a wide range of substances and matrices, including soil (Wang et al. 2025), sediments (Sun et al. 2025), anaerobic digestates (Caroca Sepúlveda et al. 2024), and foods like fish sauces and drinks (Reichstädter et al. 2021; Senila and Angyus 2025; Senila et al. 2024). In the DGT technique, a simple plastic device holds a binding resin gel placed behind a gel diffusion layer and membrane filter, which accumulates the analytes from a solution. The device is deployed for a known time in the analyzed solution to accumulate the analyte in a controlled way. The mass of the analyte is measured in the binding gel and used to calculate the concentration in the analyzed solution (Davison and Zhang 2012). Even though the waiting time for immersing a DGT device in the solution to be analyzed is quite long, the actual working time of the analyst is reduced, making it overall easy to run in routine laboratories.

In general, ionic strength does not strongly affect the accumulation of bivalent trace metal cations by the Chelex 100 binding resin layer, which has high selectivity toward these cations, ensuring their extraction and matrix separation. DGT has been reported to exhibit satisfactory performance even in complex matrices, such as seawater, which contain high concentrations of sodium ions (Cindrić et al. 2020; Gao et al. 2019; Tankéré‐Muller et al. 2012). Consequently, our hypothesis was that the DGT technique could be used to minimize matrix effects in the determination of Cd and Pb in table salt by extracting these elements form salt dissolved in aqueous solution before spectrometric measurement. However, the development and validation of new analytical methodologies proposed for the control of contaminants in food, that meet the requirements set out in Commission Decision (2002/657/EC) and Commission Regulations (2011/836/EU; 2007/333/EC) represents one of the most significant challenges. This paper presents an analytical methodology for the application of the DGT technique as a matrix separation and preconcentration tool for the determination of Cd and Pb in table salt. While DGT has primarily been used in environmental samples to measure the “labile” fraction of analytes, this study aims to determine the total concentration, which is expected to correspond to the “labile” fraction due to likely absence of significant complexation in the sample. The paper has three main objectives: (1) to develop passive sampling methods for Cd and Pb in salt solutions by evaluating linear accumulation, diffusion coefficients, preconcentration factors, and sample matrix composition after elution; (2) to validate the method against European standards for food control analytical methods; and (3) to analyze table salt from Romania markets to assess human health risks from Cd and Pb ingestion.

## 2 Experimental

### 2.1 Reagents, Reference Materials, and DGT Samplers

Single‐element Cd and Pb standards (1000 mg L^−1^; Merck) were used for GFAAS calibration and solution preparation. Multi‐element ICP standard IV (23 elements, 1000 mg L^−1^; Merck) was used for ICP‐OES calibration. Ultrapure 60% HNO_3_ (Merck) was used for salt digestion and to prepare the 1 mol L^−1^ HNO_3_ elution solution for Chelex‐100 resin gel. Ultrapure water (18.2 MΩ cm) was produced using a Purelab Flex 3 system (ELGA LabWater).

The highest concentration standards from each GFAAS calibration curve—10 μg L^−1^ Cd and 50 μg L^−1^ Pb—were prepared by diluting 1000 mg L^−1^ stock solution with 1 mol L^−1^ HNO_3_. Subsequently, the calibration standards were automatically diluted using the GFAAS autosampler. For GFAAS measurements, matrix modifiers comprising 10% NH_4_H_2_PO_4_ and 1% MgNO_3_ (both obtained from Perkin‐Elmer) were used. A blank solution consisting of 0.2% NaCl (p.a.; Sigma Aldrich, Germany) in ultrapure water was utilized.

Standard plastic DGT holders with an exposed window area of 3.14 cm^2^, diffusive gels (0.8 mm thick, 15% acrylamide, 0.3% agarose‐derivative crosslinker) and resin gels (Chelex 100 type, iminoacetate functional group) were acquired from DGT Research Ltd. (https://www.dgtresearch.com/). Polyethersulfone filter membranes (0.45 μm pore size, 0.12 mm thick) were used in each DGT unit to cover the diffusive gels. The assembly of DGT units was carried out in clean laboratory conditions under a flux of filtered air (clean room Class ISO 8).

### 2.2 Instrumentation

Cd and Pb concentrations in elution solutions (1 mol L^−1^ HNO_3_) from DGT resin gels were determined using a Perkin‐Elmer PinAAcle 900T GFAAS (Norwalk, USA) equipped with specific Electrodeless Discharge Lamps (EDL) operated at the wavelengths of 228.80 and 283.31 nm, respectively. For Cd determination, the following heating program was applied: 130 for drying, 500 for pyrolysis, 1500 for atomization, and 2450°C for cleaning. For Pb determination, the heating program was as follows: 130 for drying, 850 for pyrolysis, 1600 for atomization, and 2450°C for cleaning. Sample aliquots of 20 μL were injected into the graphite tube along with 5 μL of matrix modifiers (NH_4_H_2_PO_4_ + Mg(NO_3_)_2_), selected according to the manufacturer's recommendation. Calibration curves were constructed over the ranges of 0–5 for Cd and 0–40 for Pb μg L^−1^, respectively. Three replicate determinations were performed for each analysis using GFAAS. Solution pH was measured using a pH meter (model S400) from Mettler Toledo.

### 2.3 Determination of Cd and Pb Content in Refined and Unrefined Table Salt

Ten types of refined and unrefined table salt from Romanian markets (S1–S10) were analyzed in this study. For the determination of Cd and Pb contents in the salt samples, 2 g of sample was placed into a glass beaker, followed by the addition of 50 mL of ultrapure water and 5 mL of HNO_3_ 60%, to eliminate carbonates and prevent possible precipitation. The mixture was heated on a sand bath until nearly dry, and then the residue was dissolved in ultrapure water and diluted to 1000 mL. All vessels used were presoaked in a 10% (v/v) HNO_3_ solution overnight and rinsed with ultrapure water before use. Blank solutions consisting of elution solutions from DGT devices exposed to 0.2% NaCl were used. Three replicate measurements were performed to ensure quality control.

### 2.4 Estimation of Effective Diffusion Coefficients in Salt Solutions

The effective diffusion coefficients (*D*_e_) for Cd and Pb were calculated by conducting experiments on the linear accumulation of Cd and Pb based on DGT deployments over a 24‐h period (Diviš et al. 2020). Ten DGT devices were suspended using nylon fishing line to prevent contact between the diffusion surface and the vessel walls and deployed in 1 L of a 0.2% NaCl solution (pH = 5) spiked with 100 μg L^−1^ Cd and Pb. At this spiking level, the amount of metals removed by the 10 DGT devices during uptake can be considered negligible, corresponding to approximately 0.2% of the added concentration. The feed solution was introduced in a high‐density polyethylene container (pre‐cleaned with 10% [v/v] HNO_3_ solution and washed with ultrapure water), a material considered relatively inert, to prevent the adsorption of Pb and Cd onto the container walls. The solutions were maintained at 22°C ± 2°C and stirred at 300 rpm during the deployment. Two DGT devices were removed from the solution after 2, 4, 6, 12, and 24 h, and the binding gel was immersed in a centrifuge tube for elution with 1 mL of 1 mol L^−1^ HNO_3_ for approximately 24 h at room temperature. The eluate was analyzed for Cd and Pb concentrations using GFAAS, and the mass (*M*) of each metal accumulated on the resin, in ng, was calculated using Equation (1) (Pelfrêne et al. 2011):

(1) $$M=\frac{C_{e}\times \left(V_{g}+V_{e}\right)\times \mathrm{DF}}{f_{e}}$$

where *V*_e_ is the volume of the eluent (1.0 mL), *V*_g_ is the volume of resin gel (0.15 mL), DF is the dilution factor (1, in this study, the elution solutions were directly analyzed by GFAAS), and *f*_e_ represents the elution factor (0.85) (DGT Research Ltd., Lancaster, UK, https://www.dgtresearch.com/).

The effective diffusion coefficient (*D*_e_) values were determined from the slope (*k*) of linear accumulation curves by plotting the accumulation of metal, normalized to its concentration in solution, over various deployment durations (calculated via mass balance), as a function of time. Equation (2) (Reichstädter et al. 2020) was used to calculate *D*_e_, based on the slope *k* and the metal concentration *c* in the feed solution.

(2) $$D_{e}=\frac{k\times \Delta g}{A}$$

here, Δ*g* represents the diffusive media thickness (0.8 mm diffusive gel + 0.14 mm filter, whereas the diffusive boundary layer [DBL] was neglected), and *A* represents the exposure area (3.14 cm^2^).

The DGT concentration of metals were calculated using Equation (3) (Zhang and Davison 2015):

(3) $$C_{\mathrm{DGT}}=\frac{M\times \Delta g}{t\times A\times D_{e}}$$

where *t* represents the immersion time.

The influence of pH on the metal uptake was evaluated by immersing six DGT devices into the feed solution containing 0.2% NaCl and 100 μg L^−1^ Cd and Pb at different pH values of 4, 5, and 6 for 4 h. The measured *c*_DGT_ concentrations and the concentrations measured directly in the feed solutions (*C*_SOL_) were used to calculate recovery: *R* = *C*_DGT_/*C*_SOL_. In a similar manner, the influence of NaCl concentration in the feed solutions was tested. In this case, the DGT devices were immersed in feed solution with three different levels of NaCl concentrations: 0.2%, 1%, and 5%, and recovery was calculated in the same way.

### 2.5 In‐House Validation of the DGT Method for Cd and Pb Content Preconcentration Prior to GFAAS Determination

The applicability of the DGT method was assessed by considering the maximum admissible levels (MAL) of Cd and Pb established in Commission Regulation ([EU] 2023/915). The method was validated according to the requirements outlined in Commission Decision (2002/657/EC) and Commission Regulations (2011/836/EU; 2007/333/EC), which govern analytical methods used by official laboratories for food control. Validation parameters included limits of detection and quantification (LOD and LOQ), repeatability, trueness, and measurement uncertainty.

The LOD was estimated using the 3*σ* criteria (LOD = 3 × *σ*), according to International Union of Pure and Applied Chemistry (IUPAC) recommendations by measuring the mass of analyte accumulated in five different DGT devices following deposition in a 0.2% NaCl solution (prepared using NaCl p.a., Sigma Aldrich, Germany and ultrapure water), and standard deviations (*σ*) of these masses in ng. The LOD was then converted to concentration (μg L^−1^). The LOQ was considered as two times the LOD. The average of the blanks was not taken into consideration since its value was below than three times the standard deviation of the blanks.

Since no Certified Reference Material (CRM) with certified content of Cd and Pb in table salt was found on the market, trueness was assessed using spiked solutions. At mg kg^−1^ concentration levels, a method is considered valid if the measured content remains within the limit −20% to +10% of the target value, according to Commission Decision (2002/657/EC). Repeatability was evaluated in terms of relative standard deviation of repeatability (RSD_r_), calculated by analyzing six aliquots of fortified samples in parallel at concentrations levels of 0.2 and 1 times, respectively, the Residual Maximum Limit (RML). The observed RSD_r_ was compared with the predicted standard deviation (PRSD) estimated using Horwitz’ equation (Horwitz et al. 1980):

(4) $$\text{PRSD}=2^{\left(1-0.5\log C\right)}$$

where *C* is the maximum mass fraction of Cd and Pb in table salt expressed as a mass fraction.

The repeatability meets the criteria of Commission Decision (2007/333/EC) and Commission Regulation (2011/836/EU) if the HorRat index (*R*), assessed as the RSD/PRSD ratio, is < 2. The combined standard measurement uncertainty (*U*) must be below the maximum standard measurement uncertainty (*U*_f_) calculated using Equation (5):

(5) $$U_{f}=\sqrt{{\left(\frac{\mathrm{LOD}}{2}\right)}^{2}+{\left(\mathrm{\alpha C}\right)}^{2}}$$

where LOD is the limit of detection, *C* is the concentration of interest, and *α* is a numeric factor to be used depending on the value of *C* (*α* = 0.18 for concentrations between 51 and 500 μg kg^−1^, and 0.15 for concentrations between 501 and 1000 μg kg^−1^) (Commission Regulation 2011/836/EU).

The DGT measurements were validated against an independent analytical technique, in our case ICP‐OES, using a Perkin‐Elmer Optima 5300DV spectrometer to assess the influence of chloride complexation and potential deviations arising from differences in diffusion coefficients and species lability. Two salt samples, S1 and S2, were dissolved as described in Section 2.3 and then analyzed in parallel by DGT, followed by GFAAS, and directly by ICP‐OES, respectively. Because the Cd concentration was below the limit of detection (LOD), 20 μg L^−1^ of Cd was added to the dissolved sample solutions, corresponding to a Cd concentration of 5000 μg kg^−1^ in the solid salt samples.

### 2.6 Estimation of Potential Human Health Risk Posed by Cd and Pb Ingestion

The noncarcinogenic risk to human health posed by Cd and Pb content in salt samples was calculated as the Hazard Quotient (HQ) for each element, according to Equation (6). The hazard Index (HI) indicates the overall risk associated with the sum of HQ for Cd and Pb (Giri et al. 2022).

(6) $$\mathrm{HQ}=\frac{\mathrm{PTE}\times \mathrm{IR}\times \mathrm{ED}\times \mathrm{EF}}{\mathrm{AT}\times \mathrm{BW}\times R_{f}D}$$

where PTE is the content of potentially toxic element (Cd or Pb) (mg L^−1^) in the salt sample and IR represents the average ingestion rate of salt (12.9 g day^−1^ in Romania according to the World Population Review 2026). The exposure duration (ED) was considered 56 years, and the exposure frequency (EF) was set to 365 days year^−1^. AT and BW represent the average time of exposure (20,440 days) and the average body weight (70 kg), respectively. The reference dose (*R*_f_ *D*) for each ingested PTE was taken from the United States Environmental Protection Agency tables, as 0.001 Cd, and 0.0035 Pb mg kg^−1^ day^−1^ (Atamaleki et al. 2020). If HQ < 1.0, there are no noncarcinogenic risk effects, whereas HQ > 1.0 indicates possible adverse effects (Sabala et al. 2024).

## 3 Results and Discussion

### 3.1 Uptake Kinetics and Effective Diffusion Coefficients Through Resin Gel in 0.2% NaCl Solution

**FIGURE 1:**
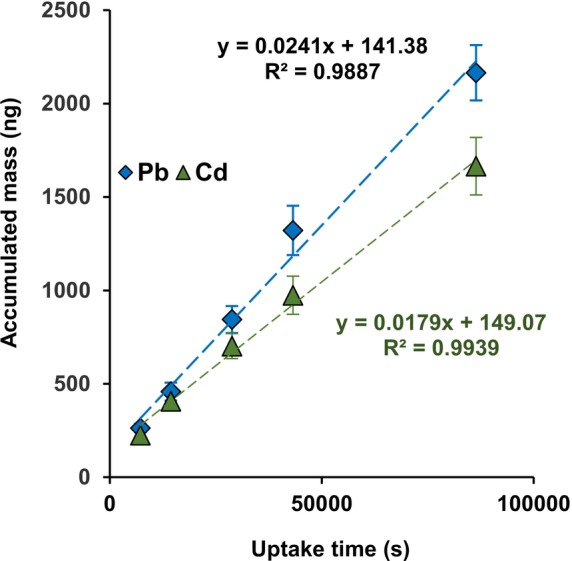
Uptake kinetics of Cd and Pb by DGT from a 0.2% NaCl solution (100 μg L^−1^ Cd and Pb, pH = 5). Deployment conditions: Temperature = 22°C ± 2°C; 10 DGT units deployed in 1 L of source solution, stirring rate = 300 rpm. DGT units retrieved after 2, 4, 8, 12, and 24 h. Elution performed with 1 mL of 1 mol L^−1^ HNO_3_. Error bars represent the standard deviation (*n* = 2).

The effective diffusion coefficients (*D*_e_) for Cd and Pb through the diffusive gel in 0.2% NaCl solution were determined by performing standard DGT testing procedures based on the linear accumulation of the analyte as a function of time. The mass of both Cd and Pb accumulated linearly over a deployment period of 24 h (Figure 1). The RSD for the analysis of parallel DGT deployments was < 10% in all cases. The coefficients of determination (*R* ^2^) were 0.9939 and 0.9887, respectively, indicating proper DGT function.

The effective diffusion coefficients of Cd and Pb in 0.2% NaCl solution were 5.37 × 10^−6^ cm^2^ s^−1^, and 7.22 × 10^−6^ cm^2^ s^−1^, which represent 0.98 and 0.96 of the value of diffusion coefficients at 22°C (5.61 × 10^−6^ cm^2^ s^−1^ and 7.40 × 10^−6^) as recommended by the DGT producer (DGT Research Ltd., https://www.dgtresearch.com/). This confirms that DGT can accurately accumulate Cd and Pb from salt solution. The obtained *D*_e_ values were further used to assess method performance parameters and for Cd and Pb determination in real samples.

### 3.2 The Influence of pH and NaCl Concentration on Metals Accumulation

**FIGURE 2:**
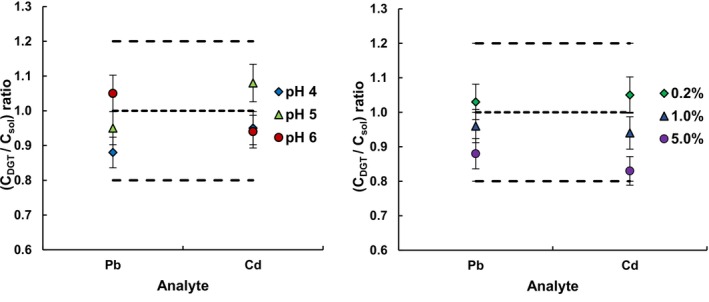
(a) Effect of pH on the uptake of Cd and Pb by Chelex‐100 binding gel. Deployment conditions: 100 μg L^−1^ Cd and Pb in 0.2% NaCl solution, pH = 4, 5, 6, temperature = 22°C ± 2°C, deployment time = 24 h. Error bars represent the standard deviation (*n* = 2). (b) Effect of NaCl concentration on the uptake of Cd and Pb by Chelex‐100 binding gel. Deployment conditions: 100 μg L^−1^ Cd and Pb in 0.2%, 1%, 5% NaCl solution, pH = 6, temperature = 22°C ± 2°C, deployment time = 24 h. Error bars represent the standard deviation (*n* = 2).

The typical method for evaluating the effect of pH and ionic strength (NaCl concentration) on metal accumulation involves exposing DGT samplers to synthetic solutions with known pH and ionic concentrations. The obtained results are displayed in Figure 2.

The literature data indicates that the pH of the solution can alter the structural configuration of the functional groups within the resin used in the binding gel, potentially affecting its retention capacity (Atzei et al. 2001). As shown in Figure 2a, the results of our experiments indicated that both Cd and Pb are retained with satisfactory performance within the pH range of 4.0 and 6.0, the *R*‐value ranging from 0.88 to 1.05 for Pb and 0.94 to 1.08 for Cd. Given that pH values below 4 are generally not recommended for DGT deployment (Gimpel et al. 2001; Pommier et al. 2021) and considering that acid digestion is applied in the digestion of salt samples, adjusting the pH to 5 is sufficient to ensure consistent results.

It has been already shown in the literature that the binding capacity of Chelex resin gel for Hg can be affected by increased chloride content in uptake solutions (Diviš et al. 2010). In the present study, *R*‐values ranged from 0.88 to 1.03 for Pb and 0.83 to 1.05 for Cd, indicating good agreement between *c*_DGT_ and *c*_SOL_. Because of these characteristics, DGT with Chelex gel was successfully applied for passive sampling in complex matrices such as marine environments (Cindrić et al. 2020; Gao et al. 2019; Gaulier et al. 2019). Nevertheless, the highest *R*‐values observed in this study corresponded to *c*_DGT_ measurements in solutions with a NaCl concentration of 0.2%. If a significant fraction of Cd or Pb is present as chloride complexes (e.g., CdCl^+^, CdCl^2+^, and PbCl^+^), and the diffusion coefficients of these chloride complexes differ from those of the free metal ions (typically higher for the free ions than for larger complexes), then a simple calibration based solely on the free metal ion is no longer strictly valid. Therefore, calibration should be performed using an effective diffusion coefficient (*D*_e_) determined under chloride concentrations comparable to those of the analyzed solution.

### 3.3 Performance Parameters for In‐House Validation of the DGT Method

The methodology based on DGT sampling and preconcentration prior to GFAAS determination was validated in terms of LOD, LOQ, repeatability, trueness, and measurement uncertainty, considering the requirements outlined in Commission Decision (2002/657/EC) and Commission Regulations (2011/836/EU; 2007/333/EC). The LODs and LOQs values, estimated using the 3*σ* criteria by deploying five DGT devices in 0.2% NaCl solution (blanks) followed by GFAAS determination, are presented in Table 1.

**TABLE 1:** Performance parameters (LOD, LOQ) for the determination of Pb and Cd in salt for a deploying period of 24 h.

Performance parameter	*C*_DGT_ Cd (μg L^−1^)^a^	*C*_DGT_ Pb (μg L^−1^)^a^	*C*Cd (μg kg^−1^)^b^	*C* Pb (μg kg^−1^)^b^
LOD	0.08	0.16	38	81
LOQ	0.15	0.32	77	162

^a^ Calculated considering the instrumental LOD or LOQ and a 24‐h deployment time at 22°C.

^b^ Calculated considering the digestion protocol based on 2 g table salt dissolved to a final volume of 1000 mL.

The acceptance criteria for Pb and Cd determination in foodstuffs, according to Commissions Regulations (2007/333/EC; 2011/836/EU), are that the LOD should be one‐tenth of the maximum level and LOQ one‐fifth of the maximum level in salt. For both Cd and Pb, the LODs and LOQs were well below the values required for acceptance, considering the maximum levels of 500 and 1000 μg kg^−1^, respectively. Moreover, in DGT passive sampling, the LOD and LOQ, expressed as *c*_DGT_, are dependent on the deployment time, as the mass of analyte accumulated in the binding layer increases with exposure duration. According to Equation (3), *c*_DGT_ is inversely proportional to deployment time. For example, doubling the deployment time results in a twofold decrease in both LOD and LOQ, assuming all other parameters remain constant.

Trueness was evaluated by analyzing spiked solutions at concentration levels of 0.2 and 1 times the Residual Maximum Limit (RML), corresponding to 100 and 500 μg kg^−1^ for Cd, and 200 and 1000 μg kg^−1^ for Pb, respectively. Considering the digestion protocol (0.2 g of salt dissolved in 100 mL of solution), the corresponding solution concentrations were 0.2 and 1 μg L^−1^ for Cd, and 0.4 and 2 μg L^−1^ for Pb, respectively. The uptake solutions were prepared in 0.2% NaCl, and in each solution, six parallel DGT samplers were introduced for 24 h at 22°C. Repeatability was evaluated using the same solutions as those used for trueness, at concentration levels of 0.2 and 1 times the RML, respectively. The results are summarized in Table 2.

**TABLE 2:** Trueness and precision for Cd and Pb determination by DGT coupled with GFAAS at concentration levels of 0.2 and 1 times the RML in table salt (*n* = 6 parallel determinations).

Analyte	Theoretical concentration in salt (μg kg^−1^)	Theoretical *C*_DGT_ (μg L^−1^)	*X* ± SD (μg L^−1^)	RSD_r_ (%)	*R* (%)
Cd	500	1	0.90 ± 0.10	11.0	90
	100	0.2	0.17 ± 0.02	13.1	85
Pb	1000	2	1.83 ± 0.23	12.3	92
	200	0.4	0.37 ± 0.05	12.6	92

*Note:* *X*—average value.

Abbreviations: *R*, recovery (%); RSD_r_, relative standard deviation for repeatability; SD, standard deviation.

The observed RSD_r_ was compared with the predicted standard deviation (PRSD) estimated using Horwitz's equation. PRSD calculated for Cd, at a maximum admitted concentration of 500 μg kg^−1^, is about 17.7%, while for Pb, at a maximum admitted concentration of 1000 μg kg^−1^, the PRSD is about 16.0%. HorRat index values, calculated as observed RSD/PRSD, were 0.62 for Cd and 0.77 for Pb, indicating acceptable precision (HorRat index < 2).

At mg kg^−1^ concentration levels, a method is considered valid if the measured content remains within the limit of −20% to +10% of the target value (Commission Regulation 2011/836/EU). As shown in Table 2, recoveries were in the target interval, thus the method trueness meets the terms of Commission Regulation 2011/836/EU.

The combined standard measurement uncertainty (*U*) was determined using the law of uncertainty propagation. The sources of uncertainty included standard deviations from repeated measurements, gel thickness tolerances (±0.03 mm), temperature corrections (±2°C), device geometry tolerances (exposure area ±0.14 cm^2^), and variations in deployment time (±300 s), estimated according to information reported by Warnken et al. (2006). The estimated *U* values were 13.2% for Cd and 14.0% for Pb, corresponding to 66 μg kg^−1^ for Cd and 140 μg kg^−1^ for Pb when calculated at the RML. These values were below the maximum standard measurement uncertainty (*U*_f_), determined using Equation (5), which was 90 μg kg^−1^ for Cd and 150 μg kg^−1^ for Pb. Accordingly, the method complies with the provisions of Commission Regulation 2011/836/EU, including those related to measurement uncertainty.

To assess matrix separation efficiency, concentrations of major cations (Na, Ca, K, and Mg) were determined by ICP‐OES in a feed solution containing 0.2% table salt and in the eluate from DGT resin. The percentages of remaining major cations from the initial solution were 0.1%, 3.3%, 0.3%, and 2.7% for Na, Ca, K, and Mg, respectively, indicating the excellent matrix separation capability of the DGT tool. This significantly reduces the interference of salts and provides solutions that are easier to be analyzed by spectrometric methods.

### 3.4 Determination of Cd and Pb Content in Table Salt

Ten types (S1–S10) of refined and unrefined salts from Romanian markets were analyzed using DGT passive sampling and GFAAS measurement. The concentrations of Cd and Pb in the salt samples are presented in Table 3.

**TABLE 3:** Results (mean ± *U*) for Cd and Pb in refined and unrefined table salt, *n* = 3 parallel determinations using DGT passive sampling and GFAAS determination.

Sample	Salt type	Cd (μg kg^−1^)	Pb (μg kg^−1^)
S1	Unrefined rock salt	< 77	4870 ± 681
S2	Unrefined rock salt	< 77	7860 ± 1100
S3	Unrefined rock salt	< 77	527 ± 82
S4	Refined iodized rock salt	< 77	199 ± 29
S5	Refined iodized rock salt	< 77	245 ± 34
S6	Refined iodized rock salt	< 77	< 162
S7	Refined iodized rock salt	< 77	< 162
S8	Refined iodized rock salt	< 77	584 ± 74
S9	Refined iodized salt from seawater	< 77	< 162
S10	Refined iodized salt from seawater	< 77	< 162
MAL		500	1000

*Note:* DGT concentration calculated for a deployment of DGT for 24 h at 22°C.

As shown in Table 3, Cd concentrations were below the limit of quantification (LOQ) in all analyzed samples. However, Pb was quantitatively detected in six of the 10 commercially available table salts that were analyzed. Fortunately, the Pb content did not exceed the maximum allowed limit in any of the refined salt samples (obtained from rock or seawater). In contrast, in two of the three unrefined rock salt samples commonly used in Romanian households for food preservation, the Pb content exceeded the maximum allowable level for table salt by nearly five and eight times, respectively. Elevated contamination levels observed in rock salt are often attributed to its geological provenance and the absence of purification procedures during its production. It has been previously reported that refining techniques, including recrystallization and purification stages, substantially reduce heavy metal concentrations in salt (Mohammadi et al. 2025).

Given that many salts are derived from geological deposits or evaporated seawater, trace concentrations of heavy metals may inherently exist in the raw materials prior to refinement. Furthermore, contamination can be introduced through processing machinery and environmental exposure encountered during harvesting and transportation. As a result, numerous studies have examined the concentrations of Pb and Cd in commercially available table salts from various geographic regions worldwide. A meta‐analysis encompassing several studies reported aggregated mean concentrations of 2.98 mg kg^−1^ for Pb and 0.71 mg kg^−1^ for Cd, with substantially higher levels observed in rock salt compared to sea salt and refined salt (Mohammadi et al. 2025). Di Salvo et al. (2023) reported an average Pb concentration of 7.25 mg kg^−1^ in gourmet table salts from different regions around the world. Eftekhari et al. (2014) reported that table salt samples refined by recrystallization and washing methods had mean Cd concentrations of 0.020 and 0.017 mg kg^−1^, respectively, and mean Pb concentrations of 0.309 and 0.379 mg kg^−1^, respectively. Higher Cd concentrations of 3.201, 3.010, 4.001, and 4.208 mg kg^−1^ were reported in salt samples from Iraq (Ghazi and Duij 2024). In rock salt samples from mines in Central Anatolia, Turkey, the reported average lead (Pb) content, analyzed by energy‐dispersive X‐ray fluorescence (XRF) spectrometry, was 1.4 mg kg^−1^ (Hançerlioğullari and Eyüboğlu 2022). In another study, the concentrations of Cd in table and bakery refined salts were 0.229 and 0.240 mg kg^−1^, respectively, while the concentrations of Pb were 0.852 and 1.22 mg kg^−1^ (Heshmati et al. 2014). In a study using dysprosium hydroxide coprecipitation for metal preconcentration, the concentrations of Cd in salt ranged from below the LOD to 0.50 mg kg^−1^, while Pb concentrations ranged from 0.45 to 3.36 mg kg^−1^ (Peker et al. 2007). Cadmium content in refined and unrefined table salts from Turkey, Egypt, and Greece was reported to range from < 0.14 to 0.30 mg kg^−1^, while Pb content ranged from 0.42 to 1.64 mg kg^−1^ (Soylak et al. 2008). In conclusion, the results obtained in this study are consistent with previously reported data on the content of Cd and Pb in table salt.

To validate the DGT combined with GFAAS measurements, the DGT results were compared with direct ICP‐OES measurements performed on two dissolved salt samples (S1 and S2) containing high levels of Pb. The same samples were spiked with 20 μg L^−1^ of Cd, corresponding to a Cd concentration of 873 μg kg^−1^ in the solid sample, to enable direct determination by ICP‐OES. The results are presented in Table 4.

**TABLE 4:** Results of the analyzed salt samples S1 and S2 spiked with 10 μg L^−1^ of Cd (mean ± *U*_m_).

Analyte	*C*_DGT‐GFAAS_ (μg kg^−1^)	*C*_ICP‐OES_ (μg kg^−1^)	*R* (%)
Cd—S1	4870 ± 681	4720 ± 543	103
Cd—S2	7860 ± 1100	7430 ± 854	106
Pb—S1	912 ± 120	887 ± 98	103
Pb—S2	825 ± 109	864 ± 95	95

*Note:* *U*_m_ is the expanded uncertainty in laboratory (*n* = 5 parallel measurements, *k* = 2, 95% confidence level).

As shown in Table 4, the Cd and Pb concentrations obtained from the analysis of salt samples by DGT‐GFAAS were well in agreement with the values measured by ICP‐OES. The recoveries, calculated using the ICP‐OES values as reference, ranged between 95% and 106%. The comparison of measurement results was performed by evaluating the differences between the DGT‐GFAAS and ICP‐OES values relative to the combined uncertainty of the two measurement techniques. The difference between average measured concentration (*C*_DGT‐GFAAS_) and the reference method (*C*_ICP‐OES_) was calculated using Equation (7):

(7) $$\Delta_{m}=\left|C_{\mathrm{DGT}‐\text{GFAAS}}-C_{\mathrm{ICP}‐\mathrm{OES}}\right|$$

The combined uncertainty (*U*_Δ_) was calculated using the formula:

(8) $$U_{\Delta }=\sqrt{U_{\mathrm{DGT}‐\text{GFAAS}}^{2}+U_{\mathrm{ICP}‐\mathrm{OES}}^{2}}$$

The differences between the values measured using DGT‐GFAAS and ICP‐OES were lower than the expanded uncertainty, indicating that no significant difference existed between the two methods. These results confirm the validity of the developed DGT method for table salt analysis.

### 3.5 Health Risk Assessment of Salt Ingestion

**FIGURE 3:**
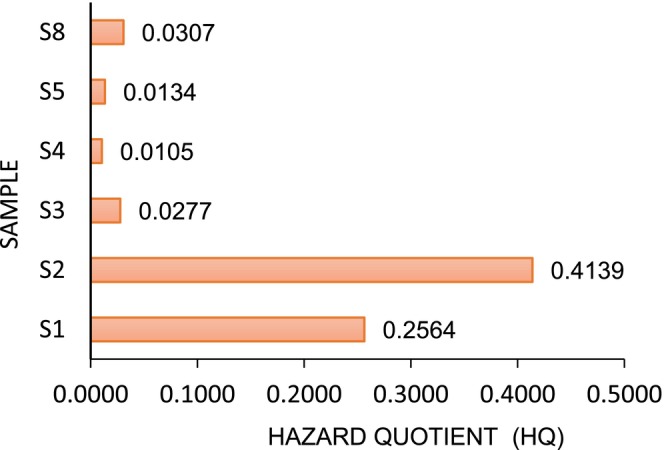
Hazard quotients, HQs, posed by salt samples consumption due to the Pb content.

When indications arise that the consumption of contaminated food may pose health risks, it is imperative to undertake a risk evaluation. This process serves as an effective method for identifying health risks related to prolonged exposure to one or more contaminants. Since the Cd concentration in all analyzed samples was below LOQ and well below MAL in table salt, its health risk was not evaluated. The noncarcinogenic risk posed by Pb content in salt samples was calculated as HQ, using Equation (6), for each sample containing Pb above LOQ. The obtained results are presented in Figure 3.

For an average ingestion rate of 12.9 g day^−1^ salt in Romania, HQs were in the range of 0.0105–0.4138, in all cases < 1.0, indicating no noncarcinogenic risk effects (Sabala et al. 2024). Nevertheless, elevated HQ values for Pb content in salt, as observed in samples 1 and 2 (unrefined rock salt), may present potential health risks to humans if consumed in quantities higher than the average ingestion rate. Previous studies have reported that, despite the inherent toxicity of Pb and Cd, various risk assessments suggest that salt accounts for only a minor proportion of the overall dietary exposure to these metals. This is primarily attributed to the relatively limited daily intake of salt compared to other food sources (Salahel din et al. 2025).

The proposed analytical procedure offers several advantages, as it is simple to perform and eliminates multiple sample pretreatment steps required for the separation and preconcentration of Cd and Pb from complex matrices such as salt. These benefits are mainly attributed to the simplicity and excellent capabilities of the DGT technique.

## 4 Conclusions

In this study, the DGT devices with Chelex‐100 resin gel were applied for the first time as preconcentration and matrix separation tools prior to GFAAS analysis for the determination of Cd and Pb in table salt. Linear accumulation of both Cd and Pb from a 0.2% NaCl was obtained, with coefficients of determination of 0.9939 and 0.9887, respectively. The effective diffusion coefficients of Cd and Pb through the APA gel, calculated from the linear accumulation experiments, were of a similar order of magnitude to those recommended by the DGT producer.

The method was validated in accordance with the criteria specified in Commission Decision 2002/657/EC and Commission Regulations 2011/836/EU and 2007/333/EC, which establish standards for analytical procedures employed by official laboratories in food control. The validation process encompassed the assessment of parameters such as limits of detection (LOD) and quantification (LOQ), repeatability, accuracy (trueness), and measurement uncertainty. All tested performance parameters met the acceptance criteria. The analysis of major cations in the feed NaCl solution and in the eluent after DGT extraction demonstrated the outstanding matrix separation capability of the DGT, which significantly reduces interferences. The validated methodology was applied to analyze 10 types of refined and unrefined table salt from Romanian markets. The coupling of DGT with GFAAS is advantageous due to its simplicity and analytical performance. It can be further applied in routine laboratories for table salt analysis, as it meets the performance criteria established by relevant regulatory requirements in this field.

## Author Contributions

**Marin Senila:** conceptualization, methodology, investigation, supervision, funding acquisition, project administration, writing – original draft, resources, validation. **Simion Bogdan Angyus:** data curation, investigation, formal analysis, writing – review and editing, software, visualization.

## Funding

This work was supported by a grant of the Ministry of Research, Innovation and Digitization, CCCDI UEFISCDI, project number PN‐IV‐P7‐7.1‐PED‐2024‐0029, within PNCDI IV.

## Conflicts of Interest

The authors declare no conflicts of interest.

## Data Availability

The data that support the findings of this study are available from the corresponding author upon reasonable request.
